# An Electrochemical Enzyme Biosensor for Ammonium Detection in Aquaculture Using Screen-Printed Electrode Modified by Gold Nanoparticle/Polymethylene Blue

**DOI:** 10.3390/bios11090335

**Published:** 2021-09-13

**Authors:** Cong Wang, Tan Wang, Zhen Li, Xianbao Xu, Xiaoshuan Zhang, Daoliang Li

**Affiliations:** National Innovation Center for Digital Fishery, China Agricultural University, Beijing 100083, China; ndfic_wc@cau.edu.cn (C.W.); Tan.wang@cau.edu.cn (T.W.); jacklee@cau.edu.cn (Z.L.); xianbao_xu@cau.edu.cn (X.X.)

**Keywords:** biosensor, glutamate dehydrogenase, methylene blue, screen-printed electrode, ammonium

## Abstract

A SPEC/AuNPs/PMB modified electrode was prepared by electrodeposition and electro-polymerization. The electrochemical behavior of reduced nicotinamide adenine dinucleotide (NADH) on the surface of the modified electrode was studied by cyclic voltammetry. A certain amount of substrate and glutamate dehydrogenase (GLDH) were coated on the modified electrode to form a functional enzyme membrane. The ammonia nitrogen in the water sample could be calculated indirectly by measuring the consumption of NADH in the reaction. The results showed that the strength of electro-catalytic current signal was increased by two times; the catalytic oxidation potential was shifted to the left by 0.5 V, and the anti-interference ability of the sensor was enhanced. The optimum substrate concentration and enzyme loading were determined as 1.3 mM NADH, 28 mM α-Ketoglutarate and 2.0 U GLDH, respectively. The homemade ceramic heating plate controlled the working electrode to work at 37 °C. A pH compensation algorithm based on piecewise linear interpolation could reduce the measurement error to less than 3.29 μM. The biosensor exhibited good linearity in the range of 0~300 μM with a detection limit of 0.65 μM NH_4_^+^. Compared with standard Nessler’s method, the recoveries were 93.71~105.92%. The biosensor was found to be stable for at least 14 days when refrigerated and sealed at 4 °C.

## 1. Introduction

Ammonia nitrogen is an important index of aquaculture water, which refers to the sum of molecular ammonia (NH_3_) and ammonium ion (NH_4_^+^) in water. Ammonia nitrogen in aquaculture water mainly comes from feces and other excrement of aquaculture animals, residual bait, plankton debris and sludge at the bottom of the pond [1,2]. When water is anoxic, denitrification of organic matter, nitrate and nitrite will also produce a part of ammonia nitrogen under the action of anaerobic bacteria. The symptoms of chronic ammonia nitrogen poisoning in aquatic animals include reduction of food intake, poor growth and tissue damage. At the same time, abnormal behaviors such as hyperactivity, swimming in the water surface layer, loss of balance or even death may occur.

The nitrogen cycle in aquaculture water is affected by physical factors and biological processes. Ammonia nitrogen is at trace level, and its concentration is easy to change. At present, the detection methods of ammonia nitrogen in aquaculture water mainly include spectrophotometry and colorimetry [3]. When spectrophotometry is used to determine the aquaculture water with high organic matter content, the error is large and the blank value is high [4]. The disadvantage of colorimetry is that the reagent consumption is large, and the toxicity is very strong [5,6]. Calcium ion, magnesium ion, sulfide and turbidity will interfere with the determination. For the fluorescence detection of ammonia, ammonia nitrogen can be reacted with o-phthalaldehyde (OPA) and sodium sulfite in an alkaline medium to form a fluorescent compound (λ_ex_/λ_em_: 363/426 nm) [7,8]. In our previous research, a homemade mini fluorometer showed high detection sensitivity [9]. However, the incubation time of the reaction solution before fluorescence analysis was too long. In addition, CDOM and amino acids in water samples would significantly affect the measurement results. The ammonia gas sensing electrode method has the advantages of high precision, strong anti-interference and fast response, which is suitable for automatic and continuous determination of ammonia nitrogen [10,11]. However, this method requires the pH value of the solution to be greater than 11. Ammonium ion selective electrode is seriously interfered by potassium ion in water [12,13]. The above two electrodes need frequent maintenance and calibration.

With the development of biotechnology and microelectronics as well as their mutual penetration and integration, the research of biosensor has developed rapidly, and ammonia nitrogen biosensors with glutamate dehydrogenase, alanine dehydrogenase and leucine dehydrogenase as enzyme catalyzed reactions have emerged one after another [14,15,16,17]. The researchers indirectly predicted the concentration of ammonia nitrogen by measuring the consumption of coenzyme NADH in enzymatic reaction. Because of the absorption spectrum of NADH at 340 nm, spectrophotometry can be used to analyze the change of NADH concentration [18]. However, when there is bromide, iodide, hydrogen sulfide and CDOM in the solution, the determination results become larger. Fluorescence analysis can inhibit the above interference, but the design of double enzyme structure is complex [19]. The electro-catalytic ammonia nitrogen biosensor mainly relies on the direct electro-catalytic oxidation of NADH. High oxidation potential can easily lead to other oxidation reactions, which ultimately affects the measurement results. It is worth noting that the above ammonia nitrogen biosensors based on enzymatic reaction cannot control the reaction temperature and cannot perform pH compensation when measuring the actual samples, so the measurement accuracy of the biosensors is not high.

Screen-printed electrode is a disposable enzyme electrode which is made by combining screen printing technology with mediator enzyme electrode [20,21]. It has the characteristics of low cost, good repeatability, fast response speed and less sample consumption. The nanomaterial modified electrode has unique advantages in improving the selectivity and sensitivity. The electron mediator can significantly promote the electron transfer from the oxidation center of enzyme to the electrode surface, reduce the redox potential and thus reduce the interference of other coexisting electroactive substances [22]. Based on the above three technologies, a new type of ammonia nitrogen biosensor was prepared. The disadvantages of the biosensors were improved by electrodeposition of AuNPs and electro-polymerization of methylene blue. Meanwhile, the thermostatic controller and pH compensation method were designed to further improve the measurement accuracy of the sensor.

## 2. Principle of Ammonia Nitrogen Detection

**Figure 1:**
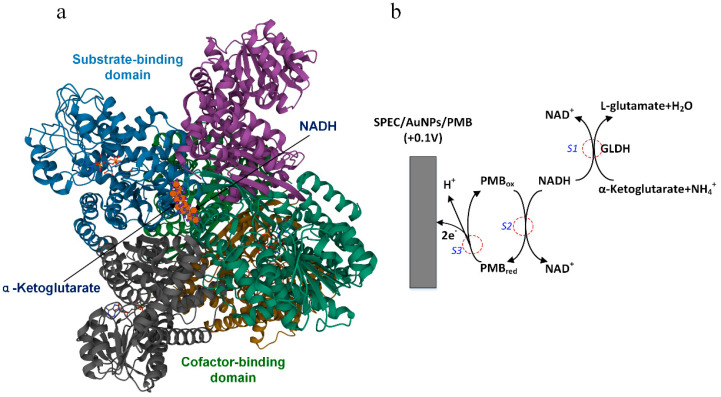
Schematic illustration of ammonia nitrogen detection. Crystal structure of glutamate dehydrogenase from Corynebacterium glutamicum (**a**). The various reactions taking place during operation of biosensor (**b**). S1—enzymatic reaction, S2—chemical reduction, S3—electrochemical re-oxidation.

Glutamate dehydrogenase (GLDH), alanine dehydrogenase (AlaDH), leucine dehydrogenase (LeuDH), phenylalanine dehydrogenase (PheDH) and valine dehydrogenase (ValDH) are the main members of the amino acid dehydrogenase family, all of which are NAD(P)+ dependent [23,24], and their enzymatic reactions are accompanied by catalytic reduction ammoniation. Amino acid dehydrogenase is derived from bacteria, fungi, plant cells and animal organs and can be purified in a laboratory. The activity of GLDH is higher than 200 U in 1 mg of solid powder, and the best activity is at pH 8.5. The optimum pH of other kinds of amino acid dehydrogenase is about 10.5, and the enzyme activity is less than 50 U/mg. The natural GLDH usually exists in the form of homologous hexamer, and its subunit structure includes substrate-binding domain and cofactor-binding domain (Figure 1a). Lysine residues are the key active sites for catalytic reaction, and they are distributed at the crack junction between the two domains. Figure 1b shows a schematic diagram of the various reactions taking place during the operation of the proposed biosensor for NH_4_^+^ determination. In the first step (S1), the enzymatic reaction is the reversible amination of α-Ketoglutarate to L-glutamate by GLDH in the presence of NADH co-factor and NH_4_^+^ ion. In the second step (S2), the remaining NADH reacts with the oxidized PMB (PMB_ox_) on the electrode surface to produce NAD^+^ and reduced PMB (PMB_red_). In the third step (S3), the electrochemical re-oxidation of PMB_red_ to PMB_ox_ involves the transfer of two electrons to the SPEC and simultaneous loss of a proton. Steps 2 and 3 constitute the electrocatalytic oxidation of NADH and provide the signal. In the absence of any NH_4_^+^ ion, the biosensor gains the maximum response because all of the NADH present participate in the electrocatalytic oxidation reaction. In the presence of NH_4_^+^ ion, the electrocatalytic current of the biosensor decreases due to the consumption of some NADH in step 1. When the concentration of substrate and the loading amount of GLDH remain constant, the decrease in current is directly proportional to the concentration of ammonium ion in the sample.

## 3. Experimental

### 3.1. Reagents

Dipotassium hydrogen phosphate, potassium dihydrogen phosphate and sodium orthophosphate were used to prepare PB solutions with different pH values. The pH value of the solution could be read directly by YSI Pro handheld instrument. Ammonium chloride needed to be dried at 100 °C for 2 h in the electric blast drying oven before preparing the standard ammonia nitrogen solution. 10 mM diphosphopyridine nucleotide (β-NADH, 98%): 7 mg β-NADH was dissolved in 1 mL ice phosphate buffer (0.05 M, pH 7.5) and stored in refrigerator for 4 °C; 80 mM α-Ketoglutarate: 12 mg α-Ketoglutarate was dissolved in 1 mL ultra-pure water, and the pH of the mixture was adjusted to 6.2~6.8 by adding a small amount of 2 M NaOH, stored in refrigerator for 4 °C. In addition, 10 mM Ammonia Nitrogen Standard Solution: 53.5 mg ammonium chloride was dissolved in 100 mL ultra-pure water and stored at room temperature; 5 mM auric chloride: 170 mg HAuCl_4_ was dissolved in 90 mL 0.05 M PB solutions, and a small amount of sodium hydroxide was pipetted to adjust the pH of the solution to 4 and stored in refrigerator for 4 °C; 10 mM methylene blue: 320 mg methylene blue was dissolved in 100 mL PBS solution and stored in refrigerator at 4 °C. All reagents except ammonium chloride do not contain ammonia nitrogen. Glutamate dehydrogenase from microorganism (L-GLDH 256 U/mg) was purchased from Yuanye organism, Shanghai, China. One unit will convert one micromole of α-ketoglutarate to L-glutamate per min at pH 8.3 at 30 °C. α-Ketoglutarate, hydroxyethyl cellulose, glycerin and ammonium chloride were purchased from Aladdin, and the purity of the reagents was AR grade.

### 3.2. Instrumentation

**Figure 2:**
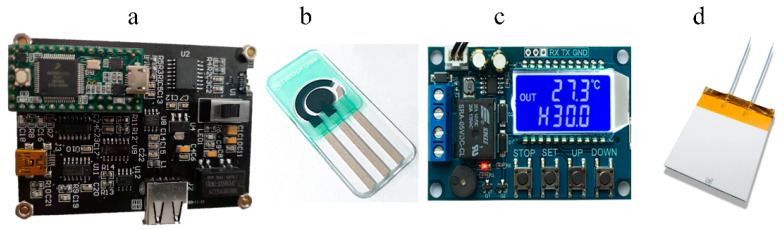
Experimental instruments. Homemade potentiostat (**a**), screen-printed electrode (**b**), homemade thermostat (**c**), ceramic heating plate (**d**).

The base transducers used in the present investigation consisted of three screen-printed electrodes (Figure 2b) deposited onto polyethylene terephthalate (PET). The working electrode consisted of an AuNPs/PMB modified screen-printed carbon electrode (SPEC/AuNPs/PMB). An Ag/AgCl electrode and a screen-printed carbon electrode served as the reference electrode and the counter electrode, respectively. The dimensions of PET substrate were 1.2 × 3.4 cm^2^, with the working electrode in the center (0.4 × 0.4 cm^2^) and the counter electrode around it. The gap between working electrode and counter electrode was 1.5 mm. In order to ensure that the liquid substrate on the working electrode would not flow to the counter electrode and the reference electrode, hydrophobic treatment was carried out between these printing electrodes. There was a height difference between the insulating coating on the surface of the screen-printed electrode and the reaction area of the electrode, which formed a reaction cell with a capacity of 50 μL.

A homemade potentiostat (Figure 2a) was used to collect the reaction signal of ammonia nitrogen biosensor with high sensitivity and speed. Potentiostat could meet the basic experiments such as cyclic voltammetry, chronoamperometry and electrode modification experiments. The potentiostat was equipped with a reference electrode interface, a counter electrode interface and a working electrode interface. The reference signal input circuit and small signal filter-amplifier circuit were encapsulated in a metal shield shell, which greatly suppressed the influence of external noise. The second-order IIR filter (Biquads) was used to obtain stable current signal.

The temperature experiments of ammonia nitrogen biosensors were carried out on a homemade mini thermostat (Figure 2c). A white multilayer alumina ceramic substrate (Figure 2d) was selected as the heating platform. The heating resistance was tungsten with 30 ohm. The dimensions of the heating plate were 4.0 × 5.0 × 0.2 cm^3^, and the working voltage was 9 Vdc. A film temperature sensor (10 k NTC, B = 3950) was fixed on the surface of the substrate with silica gel. The temperature of the heater was controlled by a circuit board embedded with PID algorithm (KP = 0.5, KI = 200, KD = 5, ΔT = 1 s). The switching time of MOS was efficiently controlled by PWM, and the control precision was ±0.2 °C.

### 3.3. Construction of Biosensor

**Figure 3:**
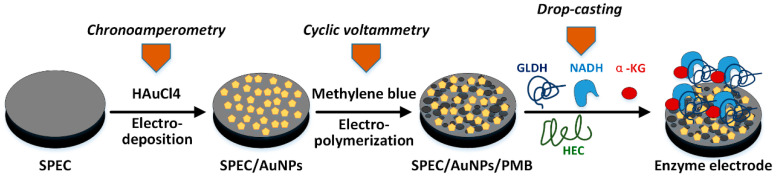
Multi step assembly process of enzyme electrode.

The printed electrode was washed in anhydrous ethanol and ultrapure water for 10 min and then dried in air. The printed electrode was placed in 5 mM HAuCl_4_ (pH = 4) phosphate buffer; the deposition potential was set at −0.2 V (vs. SCE), and the deposition time was 200 s. At this time, a layer of gold nanoparticles was deposited on the surface of the working electrode, and the residual liquid was washed out with ultrapure water. The electrodeposited screen-printed electrode (SPEC/AuNPs) was placed in methylene blue PBS solution, and the scanning window was set at −0.6~+1.2 V (vs. SCE) with several circles. The scanning rate is 100 mV/s. After scanning, the electrode (SPEC/AuNPs/PMB) was immersed in ultra-pure water for 5 min, so that the non-polymerized methylene blue molecules on the electrode surface were dissolved in water; then, the electrode was washed with ultra-pure water and stored in phosphate buffer (pH = 7.0); 25 μL of mixed substrate containing β-NADH, α-Ketoglutarate, L-GLDH and hydroxyethyl cellulose (HEC, 1.5%) was added to the surface of the working electrode (Figure 3). The volume of β-NADH in the substrate was the same as that of α-Ketoglutarate. The detection sensitivity of ammonia nitrogen biosensor was optimized by adjusting the amount of L-GLDH.

### 3.4. pH Compensation

Ammonia nitrogen in water refers to the nitrogen in the form of free ammonia and ionic ammonia. The composition ratio of the two is determined by the pH value of water. pH also affects the activity of glutamate dehydrogenase. In order to investigate the effect of pH on the whole enzymatic reaction (reductive ammoniation) system, standard samples of ammonia nitrogen were prepared in PB solution with different pH, and the calibration curves of biosensors under different pH conditions were recorded. Interpolation is an important method for approximation of discrete functions. It can be used to estimate the approximate values of functions at other points through the values of functions at a limited number of points. The principle of piecewise linear interpolation is very simple. The calculation complexity is low, and the interpolation error is small. In addition, the interpolation function has continuity.

### 3.5. Optimization of Biosensor Responses

The effects of pH and MB concentration on the electrochemical polymerization of MB were studied. The pH of the solutions was adjusted to 6, 7, 8.5, 9.1 and 9.5 by 0.05 M phosphate. The concentration of MB monomer ranged from 1 mM to 5 mM. Each solution contained 0.1 M KCl. The cyclic voltammetry was used in the electrochemical polymerization experiment. The scanning voltage range was −0.6 V~+1.2 V, and the number of scanning cycles was 20.

The loadings of NADH and α-Ketoglutarate in the substrate can directly affect the detection range and sensitivity of enzyme electrode. In order to obtain the best substrate formula, the effect of various NADH loadings on the enzyme electrode response was measured under the condition of fixed α-Ketoglutarate content (30 mM). The effect of different α-Ketoglutarate loadings on enzyme electrode response was also measured at 1.3 mM NADH content. The volume of NADH and α-Ketoglutarate in the substrate was the same, and the GLDH loading was kept constant at 2.0 U. These experiments were carried out at room temperature. Ammonia nitrogen standard solution (70 μM) was selected as the measurement sample.

By changing the loading amount of GLDH in the mixed substrate, corresponding detection range and sensitivity of biosensors can be obtained. Firstly, different concentrations of GLDH glycerol-phosphate preservation solution were prepared. Under the condition of constant concentration of NADH and α-Ketoglutarate in the mixed substrate, the same volume of GLDH was added to obtain different mixed substrate solutions. Eighteen modified screen-printed electrodes were prepared and divided into 6 groups with 3 electrodes in each group. The same substrate was added to each group. All electrodes needed to be refrigerated (4 °C) and dried for at least 12 h before use. Ammonia nitrogen standard solution (70 μM) was used as the test sample. The experiment was carried out at room temperature.

GLDH has different catalytic activities at different pH and temperature. The enzyme electrodes used in the experiment were assembled with the optimal ratio of mixed substrate solution. PB solution with different pH was used as solvent, and ammonium chloride solid powder with the same mass was used as solute for preparing a series of measurement samples. In the temperature experiment, the ceramic heating plate was controlled at different temperatures, and the effect of temperature on enzyme electrode response was monitored.

Although the ammonia nitrogen biosensor is stored in a low temperature sealed environment, with the prolongation of storage time, the substrate and enzyme assembled on the working electrode surface may leak or inactivate. In order to evaluate the shelf-life of biosensors, 18 fresh screen-printed electrodes were prepared for modification, and then, the processed enzyme electrodes were kept in cold storage. Three fresh biosensors were used to measure 300 μM ammonia nitrogen standard solution every 7 days for 6 consecutive times. The current values of each measurement were used to calculate the relative standard deviation (RSD) and response attenuation coefficient.

The effect of coexisting substances in water on the measurement of ammonia nitrogen biosensor mainly comes from the competition with substrate and inhibition or enhancement of glutamate dehydrogenase activity. Interferences with the ammonia nitrogen biosensor were investigated by using interferents such as Ca^2+^, Cu^2+^, Fe^3+^, SO_4_^2−^, Cl^−^, K^+^, NO^3−^ and EDTA. The mole ratio of coexisting substances to the concentration of ammonia nitrogen is 1:1. The amperometric response current was recorded by chronoamperometry before and after the addition of interferences, and the result was used to evaluate the interference degree of coexisting substances.

## 4. Results and Discussion

### 4.1. Electro-Polymerization of Methylene Blue (MB) Monomer

**Figure 4:**
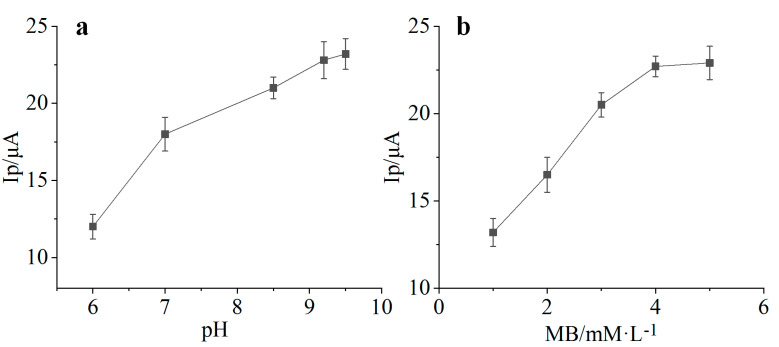
Effects of pH and MB concentration on electrochemical polymerization. Electropolymerization experiments were carried out in different MB buffer solutions. The concentration of MB was fixed at 5 mM, and the pH of the solution was adjusted to 6, 7, 8.5, 9.1 and 9.5, respectively (**a**). The concentration of MB ranged from 1 to 5 mM under pH 9.2 condition (**b**) (*n* = 3).

MB has reversible redox electrochemical behavior and is a good dielectric. The oxidation peak and reduction peak appeared at +0.2 V and −0.10 V, respectively, in the process of electrochemical polymerization. During the first 10 cycles of scanning, the peak current increased with the increase of scanning times, indicating that a layer of polymethylene blue film was formed on the surface of the working electrode. Based on the oxidation peak current of the 10th cycle, the effect of pH and MB concentration on the electrochemical polymerization performance was evaluated (Figure 4). With the increase of pH value, the oxidation peak current of PMB film increased. The concentration of methylene blue monomer solution was too low, which was not conducive to electrochemical polymerization. The oxidation peak current also increased with the increase of methylene blue monomer concentration. In the subsequent experiments for electrode modification, the concentration of methylene blue was 5 mM, and the pH of buffer solution was controlled at 9.2.

### 4.2. Characterization of Ammonia Nitrogen Enzyme Electrode

**Figure 5:**
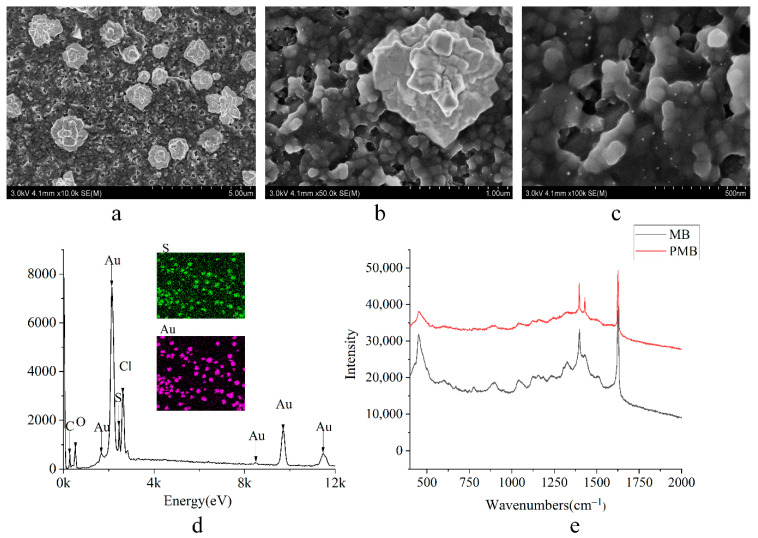
SEM images ((**a**) ×10 K, (**b**) ×50 K, (**c**) ×100 K), energy-dispersive X-ray spectroscopy (**d**) and Raman spectroscopy (**e**) of SEPC/AuNPs/PMB electrode.

The micro-zone morphologic of SEPC/AuNPs/PMB electrode was characterized by SEM. Figure 5b showed that the surface of SEPC/AuNPs/PMB electrode had obvious cluster polymer membrane. The gold nanoparticles with uniform distribution (Figure 5a) and particle size of about 800 nm appeared on the surface. Some gold nanoparticles polymerized to form a popcorn-like structure. This three-dimensional point like structure increased the specific surface area of the electrode and amplified the response current. In addition, a large number of agglomerates (Figure 5c) appeared in the gap between gold nanoparticles, which further indicated that the polymer film could be produced by electrochemical polymerization of MB on the AuNPs modified carbon electrode. The elemental distribution of the nanocomposites (Figure 5d) was further determined by the energy-dispersive X-ray spectroscopy (EDS). It clearly confirms the presence of Au, S, and Cl elements, illustrating the successful distribution of the target material. In order to further analyze the molecular structure of nanocomposites on the electrode surface, the spectral information of the PMB modified electrode and methylene blue (non-electropolymerization) were collected by Raman spectrometer (Figure 5e). It was clear that two main peaks at 1628 and 1392 cm^−1^ occurred on both red curve and black curve. The peak at 1628 cm^−1^ is attributed to phenyl ring stretching vibration. The peak at 1392 cm^−1^ is attributed to the bending vibration of N–CH_3_. After electropolymerization, an obvious peak at 1429 cm^−1^ occurred on the red curve, and this is attributed to –N(CH_3_)_2_ in poly-methylene blue. The results above indicate that the ring of methylene blue molecule was not opened after polymerization, and a new polymer membrane had been formed on the SPEC/AuNPs modified electrode.

**Figure 6:**
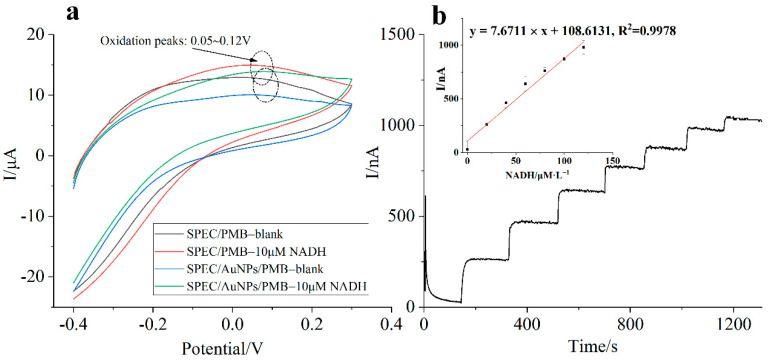
Cyclic voltammograms of SPEC/PMB electrode and SPEC/AuNPs/PMB electrode in blank and buffer solution containing 10μM NADH at a scan rate 100 mVs^−1^ (**a**), amperometric response of SEPC/AuNPs/PMB electrode to different concentrations of NADH at +0.1 V, pH 7.5 and 25 °C (**b**).

The electro-catalytic oxidation performance of PMB modified electrode for NADH plays a key role in the design of ammonia nitrogen biosensor. Figure 6a showed the cyclic voltammograms of SPEC/PMB modified electrode and SPEC/AuNPs/PMB modified electrode in the blank and buffer solution containing 10 μM NADH, respectively. It can be seen from the figure that the oxidation peak potential of NADH on both modified electrodes ranged from +0.05 V to +0.12 V, which was about 500 mV lower than that of unmodified screen-printed electrode [15,25,26,27]. Especially around +0.1 V, the oxidation peak current of PMB increased greatly in the presence of NADH due to the electrochemical re-oxidation of PMB_red_ to PMB_ox_ which involves the transfer of two electrons to the SPCE. Obviously, the response ability of the SPEC/AuNPs/PMB modified electrode to NADH is twice that of the pure PMB modified electrode. This phenomenon indicated that the composite nanostructure composed of gold nanoparticles and PMB had a good synergistic catalysis and a significant signal enhancement function.

In order to evaluate the sensitivity of the nanocomposite modified electrode for the detection of NADH, 20 μL of NADH buffer solution (10 mM) was continuously added to 10 mL deionized water for 7 times at an interval of 180 s. The working area of the electrode was completely immersed in the solution. Chronoamperometry was used in the experiment under constant stirring condition (120 rpm), and the oxidation potential was controlled at +0.1 V. It can be seen from the Figure 6b that the modified electrode had a good response sensitivity between 0~140 μM NADH. At the beginning of the experiment, the PMB on the electrode surface mainly existed in the form of oxidation state (PMB_ox_). With the progress of chemical reaction and electro-catalytic oxidation process, the content of PMB_ox_ decreased, and the electrode sensitivity to the same amount of NADH decreased simultaneously. This is why the tail of the curve became flat. In order to ensure the high measurement accuracy of ammonia nitrogen biosensor, a fresh electrode was used for each detection. The concentration of ammonia nitrogen in the sample could be calculated indirectly by measuring the consumption of NADH in the reaction system when the composition of substrate is fixed.

### 4.3. Optimization of Substrate Composition, Enzyme Loading and Temperature

**Figure 7:**
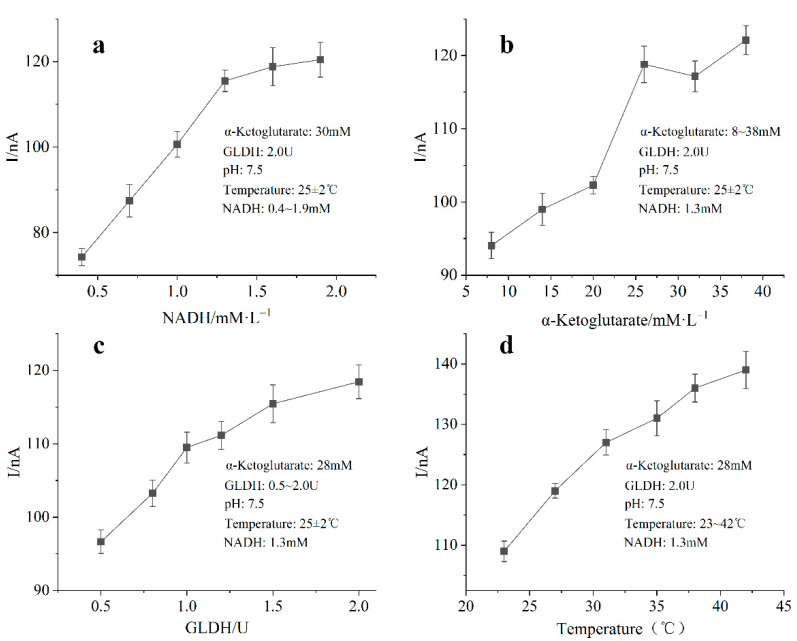
Graphs showing the response of the biosensor at different concentrations of NADH (**a**), α-Ketoglutarate (**b**) and GLDH (**c**); graphs showing the effect of temperature on the activity of bioreceptor, GLDH (**d**) (*n* = 3).

The effect of substrate concentration and enzyme loading on the detection of ammonia nitrogen biosensor was shown in Figure 7. All the four experiments were carried out with 70 μM NH_4_^+^ (pH = 7.5). When the loading of GLDH on the working electrode surface was 2.0 U and the concentration of α-Ketoglutarate was 30 mM, the concentration of NADH was adjusted from 0.4 mM to 1.9 mM. It was found that the current response of enzyme electrode increased with the increase of NADH concentration. The optimum concentration of NADH was obtained at 1.3 mM. A similar experiment was used to investigate the effect of different concentrations of α-Ketoglutarate on the enzyme electrode measurement. When the concentration of α-Ketoglutarate was lower than 28 mM, the current response intensity of enzyme electrode was positively correlated with the concentration of α-Ketoglutarate. The optimum response of the biosensor was obtained at α-Ketoglutarate concentration of 28 mM. The optimal mixed concentration of α-Ketoglutarate was 28 mM. In order to evaluate the effect of enzyme loading on the electrode reaction efficiency, the current response to the same ammonia nitrogen standard solution was detected at constant room temperature by changing the enzyme loading on the electrode surface. The results showed that the conversion efficiency of substrate and amperometric detection sensitivity of the enzyme electrode increased with the increase of GLDH loading. The substrate composition used throughout the experiments was optimized according to the above test results (NADH–1.3 mM, α-Ketoglutarate–28 mM, GLDH–2.0 U). The catalysis of enzyme is greatly affected by temperature, and the speed of enzymatic reaction can be increased by increasing the reaction temperature within a certain temperature range. The chemical essence of enzyme is protein. Too high temperature can cause denaturation of protein and inactivation of enzyme. The effect of reaction temperature on the ammonia nitrogen biosensor was measured by controlling the surface temperature of the ceramic heating plate. The results showed that the ammonia nitrogen biosensor can reduce more α-Ketoglutarate to glutamate at 42 °C. Higher temperature will prolong the heating time of the ceramic heating plate and increase the evaporation of the solution, which may affect the measurement accuracy of the biosensor. Finally, 37 °C is selected as the appropriate reaction temperature.

### 4.4. Effect of pH on the Response of Ammonia Nitrogen Biosensor

**Figure 8:**
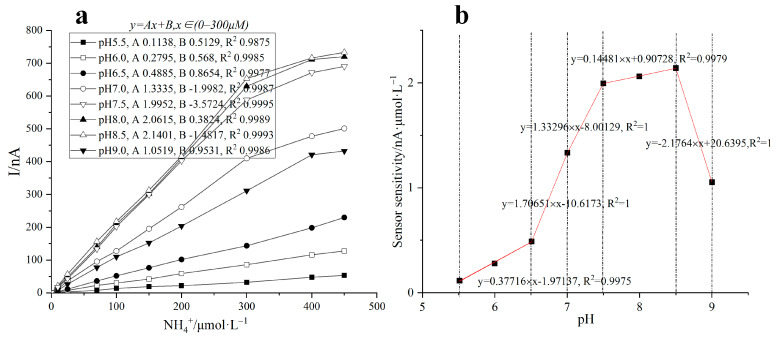
Dynamic response range of biosensor toward different concentrations of ammonium ion under different pH (**a**); Look-up table method based on piecewise linear interpolation for pH compensation (**b**). The concentrations of GLDH, α-Ketoglutarate and NADH on the enzyme electrode were 2.0 U, 28 mM and 1.3 mM, respectively. The experimental temperature was controlled at 37 °C.

pH is one of the important parameters to determine the catalytic activity of the enzyme. The change of pH can increase or decrease the enzyme activity. Different enzymes have different amino groups, so the properties of dissociated groups in enzyme molecules are different. These groups can be in different dissociation states with the change of pH. The different dissociation states of the side chain groups can affect not only the substrate binding and enzyme catalytic reaction but also the spatial conformation of the enzyme, thus affecting the catalytic activity of the enzyme. The effect of different pH on the current response of the ammonia nitrogen biosensor were investigated. As shown in Figure 8, the biosensor had a good response to ammonium ion in the range of pH 5.5~9.0, and the change value of response current increased with the increase of pH. The main reason is that under acidic and weakly alkaline conditions, ammonia nitrogen exists in the form of ammonium ion, which reacts easily with α-Ketoglutarate, and the catalytic activity of GLDH is the highest at pH 8.3. In order to further improve the detection accuracy of the biosensor, pH compensation was applied to the biosensor. Specifically, the slope values of the biosensor response curves under different pH conditions were taken as discrete samples, and a look-up table method based on piecewise linear interpolation was used to realize the rapid prediction of the sensor response sensitivity in the range of pH 5.5~9.0. The biosensor exhibited good linearity in the range of 0~300 μM. The limit of detection was about 0.65 μM (pH = 7.5) which was considered satisfactory for our purposes. To evaluate the improvement effect of pH compensation algorithm on the detection accuracy of the biosensor, the catalytic current of 70 μM ammonia nitrogen in PB solution with pH of 6.0~9.0 was tested. The results showed that there was a great difference between the results before and after pH compensation, and the compensation error was less than 3.29 μM.

### 4.5. Reproducibility, Repeatability, Long-Term Stability, Anti-Interference and Actual Sample Detection of Biosensor

**Figure 9:**
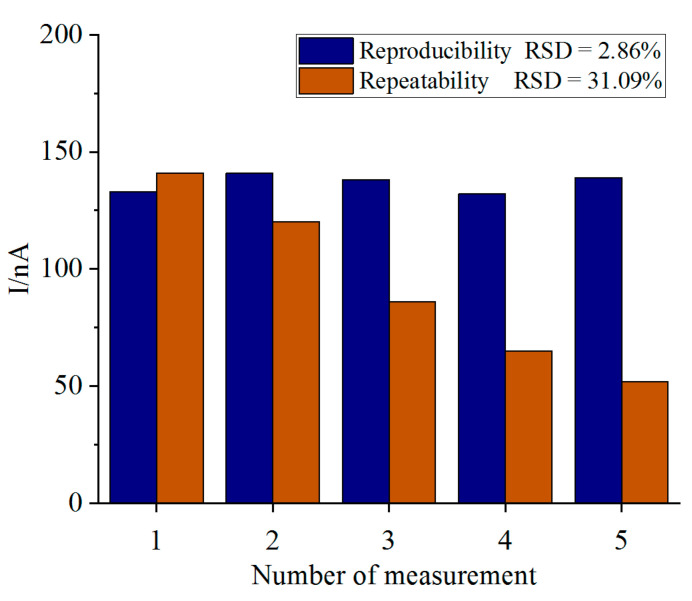
The bar graph showing the reproducibility and repeatability of the biosensor towards 70 μM NH_4_^+^ at pH 7.5.

The reproducibility and repeatability of the biosensor was evaluated at 70 μM NH_4_^+^ (pH 7.5). Six biosensors were prepared and stored at 4 °C overnight. The current difference measurements obtained using five biosensors yielded reproducibility RSD value of 2.86%. The RSD value was low enough to be considered for the high reproducibility. The same electrode was used five times at an interval of 20 min, and output currents were measured to check the repeatability of the biosensor. These measurements with the same electrode gave an RSD value of 31% (Figure 9) which was inadequate for the consideration of biosensors for reuse. By analyzing the measurement principle of the biosensor, it can be found that each measurement of the biosensor is accompanied by the consumption of mixed substrate (NADH, PMB, α-Ketoglutarate) and the loss of enzyme (GLDH) activity, which are the main reasons for the poor reusability of the electrode. In this paper, the ordinary screen-printed electrode served as the basic electrode, and the function of the biosensor was obtained by modifying nanomaterials on its surface. The preparation process was simple and low cost, which can meet the requirements of disposable use.

After functional modification and enzyme membrane immobilization, the ammonia nitrogen biosensors were placed in a sealed container for cold storage. In order to shorten the pre-activation time of the biosensors, it was necessary to place a sponge containing distilled water in the sealed container to increase the ambient humidity. During the storage of biosensors, it is inevitable that the activity of GLDH will decrease, and the reaction substrate will leak. In order to evaluate the long-term stability of ammonia nitrogen biosensors, a batch of fresh biosensors were prepared, and their stability was checked. Table 1 showed that the detection performance of the biosensors remained basically unchanged in the first 14 days. When the storage time exceeded 21 days, the output current response of the biosensors began to decrease significantly. Until the 42nd day, the output signal amplitude of the biosensors was 87% of the initial signal amplitude. The results showed that the best storage time of the biosensor is two weeks.

**Figure 10:**
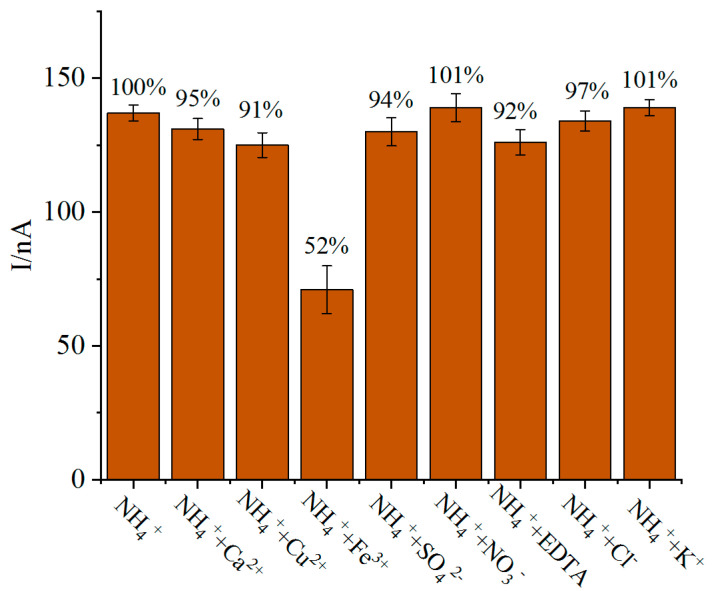
Effect of different ions (mixed solution containing 70 μM NH_4_^+^) on output current signal of biosensor (*n* = 3). All fresh biosensors were prepared under optimal experimental conditions. The experiment was carried out at 37 °C.

Because there are many kinds of anions and metal cations in natural water, they may affect the enzymatic reaction dominated by glutamate dehydrogenase. The output current signals of the biosensors before and after adding conventional disruptors were compared. According to Figure 10, trivalent iron ions inhibited the activity of glutamate dehydrogenase, and the interference of other metal ions and anions on ammonia nitrogen enzymatic reaction could be ignored. The effect of trivalent iron ions and other potential metal ions on ammonia nitrogen biosensor can be removed by adding a small amount of EDTA to the original sample.

When the ammonia nitrogen biosensor was used to measure the actual sample, a certain amount of EDTA was added to the sample. The solution was shaken to make the EDTA fully react with the metal ions in the sample, and then, the pH value of the solution was measured with a pH meter. The temperature of the ceramic heating plate was controlled at 37 °C. When the target temperature was constant, the enzyme electrode was placed on the center of the ceramic heating plate; then, 50 μL of sample was quickly absorbed and dropped onto the electrode reaction cell. The incubation time of the electrode was 60 s; then, the chronoamperometry mode was turned on, and the current difference measurement of the biosensor at 120 s was recorded. Finally, the best calibration curve of the biosensor was found according to the pH value of the solution, and the ammonia nitrogen concentration of the actual sample was calculated. In order to evaluate the detection accuracy of biosensor, the biosensor and Nessler’s reagent method [28] were used to analyze five water samples. The t values were between 1.27 and 3.62 (See Table 2) which were less than the t critical value (4.303) with 2 degrees of freedom. T-test results show that these two methods have no statistically significant difference during the determination of ammonium. Compared with the previously reported biosensors, the biosensor designed in this paper has lower detection limit and oxidation potential (See Table 3). The biosensor is more suitable for measuring low concentration of ammonia nitrogen (<50 μM) in aquaculture water. In addition, pH compensation and temperature control strategy help to improve the detection accuracy of the biosensor.

## 5. Conclusions

The disposable SPEC/AuNPs/PMB modified electrode was made for the first time, and the additions of NADH, α-Ketoglutarate and GLDH in the reaction substrate were optimized. The best storage time of the electrode was 14 days. The modified electrode shows strong concerted catalysis, high detection sensitivity and remarkable anti-interference ability. The biosensor has the advantages of low cost, small size, less sample, single use and avoiding cross interference when detecting multiple samples with the same electrode. In addition, as the technical highlights, a thermostat was designed to control the reaction temperature of the biosensor, and a pH compensation procedure based on the piecewise linear interpolation look-up table method was applied to calibrate the output signal of ammonia nitrogen biosensor in real time. The biosensor exhibited a linear range from 0.65 to 300 μM. The experimental results showed that the biosensor and Nessler’s method were basically consistent in the measurement of low concentration ammonia nitrogen samples. The biosensor can meet the requirements of rapid, highly sensitive and accurate detection of low concentration of ammonia nitrogen in aquaculture water.

## Figures and Tables

**Table 1:** The long-term stability of biosensor in 300 μM NH_4_^+^ solution at pH 7.5 (*n* = 3).

Day	Current Difference (nA)	Retained Signal (%)	RSD
1	591.25 ± 24.78	100	2.15%
7	578.54 ± 36.89	97.85	3.27%
14	572.98 ± 28.94	96.91	2.59%
21	552.05 ± 37.89	93.37	3.52%
28	538.69 ± 67.02	91.11	6.38%
42	514.51 ± 55.79	87.02	5.56%

**Table 2:** Comparison of biosensor and Nessler’s method for the detection of spiked pond water (*n* = 3).

Sample. No	Spiked Concentration Using Nessler’s Method (μM)	Spiked Concentration Using Biosensor (μM)	Recovery against Nessler’s Method (%)	T-Value
1	22.75 ± 0.10	24.10 ± 1.07	105.92%	3.47
2	39.09 ± 0.06	36.63 ± 1.87	93.71%	3.62
3	58.79 ± 0.18	61.31 ± 3.49	104.29%	1.99
4	110.63 ± 2.02	107.90 ± 5.95	97.53%	1.27
5	157.79 ± 2.64	152.11 ± 5.23	96.40%	3.01

**Table 3:** Comparison of biosensor with previously reported ammonium sensors based on dehydrogenase.

Methods	Oxidation Potential (V)	Range (μM)	PH Compensation and Temperature Control	References
SPEC/AuNPs/PMB@GLDH	0.1	0.65~300	Yes	This work
Silver epoxy-carbon WE@ f-MWCNTs + AlaDH	0.25	50~500 × 10^3^	No	[14]
SPEC@AlaDH	0.55	10 × 10^3^~100 × 10^3^	No	[15]
SPEC/AuNPs/2BME@AlaDH	0.55	100~500	No	[27]
Au/TC/AuNPs@LeuDH	0.8	20 × 10^3^~60 × 10^3^	No	[26]
SPEC/Meldola’s Blue@GLDH	0.05	2~25	No	[29]

## Data Availability

The data presented in this study are available on request from the corresponding authors.
